# Supplementary material for: Predictive Factors of Adrenal Insufficiency in Outpatients with Indeterminate Serum Cortisol Levels: A Retrospective Study
Source: Medicina (Kaunas). 2020 Jan 8;56(1):23. doi: 10.3390/medicina56010023 (PMC7022503; doi:10.3390/medicina56010023)
Supplement: Supplementary file 1 [file medicina-56-00023-s001.pdf]

Supplementary Table 1: Data categorized by history of pituitary or adrenal diseases.

Characteristic	With pituitary or adrenal diseases (*n*=240)	Without pituitary/adrenal disease (*n*=277)	P-value
**Demographic data**			
Age, (mean ±SD) (yr)	47.27±15.68	53.74±18.53	<0.001
- < 50 years old, n(%)	118 (49.17)	96 (34.66)	
- ≥ 50 years old, n(%)	122 (50.83)	181 (65.34)	0.001
Male, n(%)	128 (53.33)	124 (44.77)	0.052
Weight, (mean ±SD) (kgs)	63.37±14.74	58.20±15.64	0.002
BMI, (mean±SD)(kg/m^2)	23.00±6.46	24.76±6.12	0.001
ACTH stimulation dose, n(%)			
- 1 μg	77 (32.08)	114 (41.16)	
- 250 μg	163 (67.92)	163 (58.84)	0.033
Adrenal insufficiency, n(%)	50 (20.83)	78 (28.16)	0.054
Systolic blood pressure, (mean± SD) (mmHg)	122.69±19.49	120.26±21.19	0.178
Diastolic blood pressure, (mean± SD) (mmHg)	75.26±13.77	71.61±13.13	0.002
Underlying disease, n(%)			
- Diabetes mellitus	36 (15.00)	42 (15.22)	0.945
- Hypertension	49 (20.42)	75 (27.17)	0.073
- Chronic kidney disease	5 (2.08)	11 (3.97)	0.216
- Autoimmune disease	12 (5.00)	67 (24.19)	<0.001
- Cancer	1 (0.42)	15 (5.42)	0.001
Symptom, n(%)			
- Fatigue	24 (10.00)	83 (29.96)	<0.001
- Weight loss	4 (1.67)	22 (7.94)	0.001
- Orthostatic Hypotension	1 (0.42)	49 (17.96)	<0.001
- Nausea/vomiting	2 (0.83)	7 (2.53)	0.142
Cushingoid appearance in exogenous steroid use	2 (0.83)	64 (23.10)	<0.001
**Biochemical values**			
Serum morning cortisol (mean ±SD) (μg/dL)	9.20±3.44	9.02±3.13	0.537
< 9 μg/dL, n(%)	123 (51.25)	149 (53.79)	
≥ 9 μg/dL, n(%)	117 (48.75)	128 (46.21)	0.332
Serum basal cortisol			0.839
(mean ±SD) (μg/dL)	9.60±4.55	9.69±5.71	
< 9 μg/dL, n(%)	121 (50.42)	148 (53.43)	
≥ 9 μg/dL, n(%)	119 (49.58)	129 (46.57)	0.467
Serum K (mean ±SD) (mEq/L)	4.00±0.41	4.06±0.57	0.188
< 3 mEq/L, n(%)	5 (2.08)	32 (11.55)	
≥ 3 mEq/L, n(%)	235 (97.92)	245 (88.45)	<0.001
Serum Na (mean ±SD) (mEq/L)	137.75 (9.73)	138.84 (12.58)	0.269
< 135 mEq/L, n(%)	26 (10.83)	49 (17.69)	
≥ 135 mEq/L, n(%)	214 (89.17)	228(82.32)	0.027
Eosinophilia, n(%)	20 (11.63)	26 (11.40)	0.944
Lymphocytosis, n(%)	131 (54.58)	158 (57.04)	0.575
Serum albumin (mean±SD) (g/dL)	4.02±0.54	3.82±0.64	<0.001
< 3 g/dL	5 (2.08)	32 (11.55)	

Characteristic	With pituitary or adrenal diseases (*n*=240)	Without pituitary/adrenal disease (*n*=277)	P-value
≥ 3 g/dL	235 (97.92)	245 (88.45)	<0.001
Total cholesterol (mean±SD) (mg/dL)	188.04±43.65	177.32±55.50	0.016
< 150 mg/dL	203 (84.58)	194 (70.04)	
≥ 150 mg/dL	37 (15.42)	83 (29.96)	<0.001

Supplementary Table 2: Data categorized by primary or secondary adrenal insufficiency.

Characteristic	Primary adrenal insufficiency (*n*=18)	Secondary adrenal insufficiency (*n*=499)	P-value
**Demographic data**			
Age, (mean ±SD) (yr)	41.22±15.84	51.08±17.52	0.019
- < 50 years old, n(%)	12 (66.67)	202 (40.48)	
- ≥ 50 years old, n(%)	6 (33.33)	297 (59.52)	0.027
Male, n(%)	6 (33.33)	246 (49.30)	0.183
Weight, (mean ±SD) (kgs)	54.35±11.48	60.87±15.51	0.078
BMI, (mean±SD)(kg/m^2)	21.77±4.97	23.90±6.40	0.162
ACTH stimulation dose, n(%)			
- 1 μg	5 (27.78)	186 (37.27)	
- 250 μg	13 (72.22)	313 (62.73)	0.412
Systolic blood pressure, (mean± SD) (mmHg)	120.66±18.32	121.42±20.52	0.877
Diastolic blood pressure, (mean± SD) (mmHg)	69.99±18.03	73.43±13.25	0.290
Underlying disease, n(%)			
- Diabetes mellitus	3 (16.67)	75 (15.06)	0.852
- Hypertension	5 (27.78)	119 (23.90)	0.705
- Chronic kidney disease	0 (0.00)	16 (3.21)	0.440
- Autoimmune disease	0 (0.00)	79 (15.83)	0.067
- Cancer	0 (0.00)	16 (3.21)	0.440
Cushingoid appearance in exogenous steroid use	0 (0.00)	66 (13.23)	0.099
**Biochemical values**			
Serum morning cortisol (mean ±SD) (μg/dL)	8.67±3.61	9.12±3.27	0.678
< 9 μg/dL, n(%)	10 (55.56)	262 (52.51)	
≥ 9 μg/dL, n(%)	8 (44.44)	237 (47.49)	0.799
Serum basal cortisol			0.816
(mean ±SD) (μg/dL)	8.74±4.98	9.68±5.21	
< 9 μg/dL, n(%)	8 (44.44)	261 (52.30)	
≥ 9 μg/dL, n(%)	10 (55.56)	238 (47.70)	0.512
Serum K (mean ±SD) (mEq/L)	4.21±0.37	4.03±0.50	0.143
< 3 mEq/L, n(%)	0 (0.00)	6 (1.20)	
≥ 3 mEq/L, n(%)	18 (100.00)	493 (98.80)	0.640
Serum Na (mean ±SD) (mEq/L)	130.80 (31.86)	138.52 (9.53)	0.003
< 135 mEq/L, n(%)	3 (16.67)	72 (14.43)	
≥ 135 mEq/L, n(%)	15 (83.33)	427 (85.57)	0.791
Eosinophilia, n(%)	2 (18.18)	44 (11.31)	0.481
Lymphocytosis, n(%)	7 (38.89)	282 (56.51)	0.139
Serum albumin (mean±SD) (g/dL)	4.08±0.88	3.91±0.59	0.229
< 3 g/dL	1 (5.56)	36 (7.21)	
≥ 3 g/dL	17 (94.44)	463 (92.79)	0.789
Total cholesterol (mean±SD) (mg/dL)	169.29±50.67	182.76±50.67	0.267
< 150 mg/dL	5 (27.78)	115 (23.05)	
≥ 150 mg/dL	13 (72.22)	384 (76.95)	0.640

Supplementary Table 3: Performance capacity between serum morning and basal cortisol.

Model	ROC area	95%CI	P-value
Morning cortisol	0.65	0.60-0.70	
Basal cortisol	0.79	0.74-0.83	<0.0001

Supplementary Table 4: Multivariable risk regression of predictive factors for adrenal insufficiency clustered by ACTH dose categorized by patients with and without pituitary/adrenal diseases.

Factor	RR	95%CI	P-value
**Patients without pituitary and adrenal diseases (*n*=277)**			
Chronic kidney disease	2.32	1.63-3.31	<0.001
Cushingoid appearance in exogenous steroid and/or herbal medicine use	5.16	3.21-8.31	<0.001
Nausea and/or vomiting	1.47	1.23-1.75	<0.001
Fatigue	1.23	1.14-1.32	<0.001
Serum basal cortisol <9 μg/dL	1.97	1.87-2.06	<0.001
Cholesterol <150 mg/dL	1.38	1.19-1.60	<0.001
Serum sodium <135 mEq/L	1.20	1.12-1.30	<0.001
**Patients with pituitary and adrenal diseases (*n*=240)**			
Chronic kidney disease	5.07	1.92-13.35	0.001
Cushingoid appearance in exogenous steroid and/or herbal medicine use	4.09	2.31-7.24	<0.001
Nausea and/or vomiting	2.39	1.19-4.77	0.013
Fatigue	2.40	1.28-4.50	0.006
Serum basal cortisol <9 μg/dL	9.32	3.32-26.14	<0.001
Cholesterol <150 mg/dL	1.24	1.04-1.47	0.016
Serum sodium <135 mEq/L	0.72	0.56-0.93	0.011

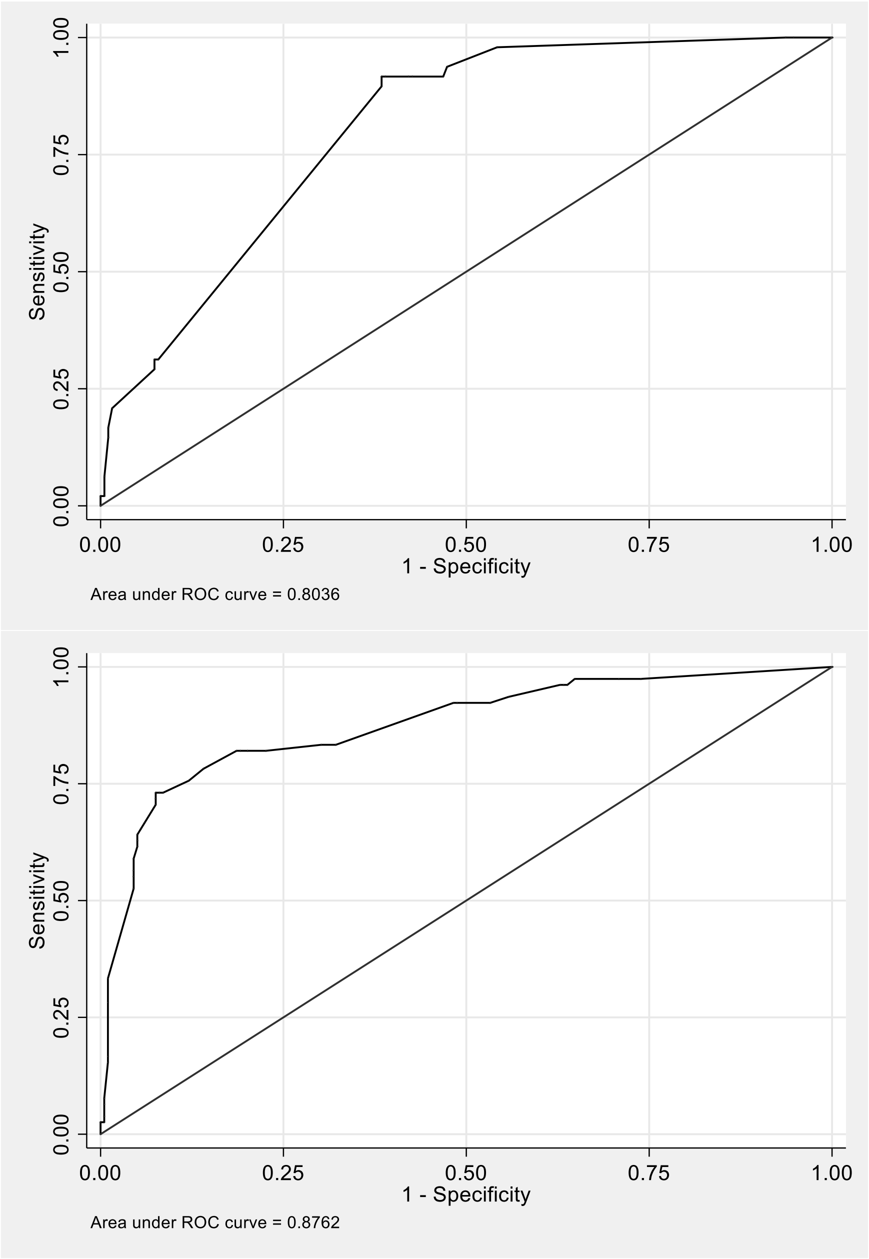

Supplementary Figure 1: Area under ROC of the predictive model for adrenal insufficiency categorized by those with or without pituitary or adrenal diseases. A) Area under ROC of the predictive model for adrenal insufficiency in patients without history of pituitary or adrenal diseases (*n*=277); B) Area under ROC of the predictive model for adrenal insufficiency in patients with history of pituitary or adrenal diseases (*n*=240).
